# Clinical Characteristics and Independent Risk Factors for Pathologic Nipple Discharge of 375 Cases

**DOI:** 10.1155/tbj/6615296

**Published:** 2025-10-28

**Authors:** Junyue Wang, Dongxiao Zhang, Qiao Huang, Na Fu, Wenjie Zhao, Yu Zhou, Yubo Guo, Xiaolong Xu, Yudong Li

**Affiliations:** ^1^Department of Galactophore, Beijing Hospital of Traditional Chinese Medicine, Capital Medical University, Beijing, China; ^2^School of Clinical Medicine, Beijing University of Chinese Medicine, Beijing, China; ^3^Clinical Diagnosis, Treatment and Research Center of Sepsis, Beijing Institute of Chinese Medicine, Beijing, China

**Keywords:** benign neoplastic lesions, breast cancer, non-neoplastic lesions, pathologic nipple discharge, precancerous lesions

## Abstract

**Background:**

While the characteristics of pathologic nipple discharge (PND) are well documented in the literature, comparative clinical and risk factor analyses across different pathologic subtypes are lacking.

**Methods:**

Medical records of patients with nipple discharge were retrospectively retrieved from an electronic medical record database and analyzed. In this study, 375 patients with a postoperative pathologically confirmed diagnosis of PND were included.

**Results:**

Age serves as an important independent risk factor for precancerous lesions and breast cancer, with the median age increasing alongside the severity of the pathology. Individuals under 45 years of age predominantly exhibited non-neoplastic and benign neoplastic lesions, whereas those over 45 were more likely to have precancerous lesions or breast cancer, with statistical significance (*p* < 0.01). Discharge color was a significant factor in distinguishing between different pathological findings (*p* < 0.01). Discharge color serves as an important independent risk factor for breast cancer. Bloody discharge was associated with a significantly higher incidence of breast cancer and precancerous lesions compared to non-bloody discharges. Upon dividing bloody discharge into brown and bright red for in-depth analysis, no significant difference was observed among the different pathological types (*p* > 0.05). Ductoscopy has a higher diagnostic rate for breast cancer and precancerous lesions (*p* < 0.01).

**Conclusion:**

These results suggest the clinical characteristics of PND patients across four pathological types: non-neoplastic lesions, benign neoplastic lesions, precancerous lesions, and breast cancer, at the same time emphasizing the importance of age and discharge color as independent risk factors in the prognosis and management of nipple discharge.

## 1. Introduction

Nipple discharge is a relatively common occurrence in females, ranking as the third most frequent breast symptom prompting medical care, following breast pain and palpable mass [1]. Notably, pathologic nipple discharge (PND) primarily manifests as spontaneous, typically single-duct, unilateral discharge, which can be clear, serous, or bloody [2, 3]. This contrasts with lactational nipple discharge, a normal milk production, and physiologic nipple discharge, characterized by bilateral, milky, green, or yellow fluid from multiple duct openings, often linked to nipple stimulation [4].

Mammography, ultrasonography, MRI, and other breast imaging techniques, along with ductoscopy, are now widely used for diagnosing and treating PND [5]. Ductoscopy, especially effective for central intraductal lesions, is a highly sensitive diagnostic method for detecting lesions in nipple discharge cases not identifiable by ultrasound or mammography [6]. A meta-analysis revealed that ductoscopy has a sensitivity of 94% and a specificity of 47% [7, 8]. Additionally, cytological examination of nipple discharge exfoliation and quantitative multiplex methylation-specific PCR (QM-MSP) serve as other diagnostic methods [9, 10].

PND may arise from benign or malignant causes, with the final diagnosis of its etiology confirmed through surgical excision of the involved duct(s) and the pathological lesion [11, 12]. For instance, intraductal papilloma or papillomatosis occurs in about 35%–48% of PND cases [13], ductal ectasia in roughly 6%–59% [5, 14], while an underlying malignancy is present in 4.4%–33% of cases [15, 16]. Nipple discharge associated with malignant lesions is not uncommon, and there is significant variation among different pathological types [17]. These lesions include carcinoma in situ and invasive carcinoma [18], with ductal carcinoma in situ (DCIS) being the most commonly reported [19]. Additionally, conditions such as intraductal papilloma, sclerosing papilloma, and atypical intraductal hyperplasia in patients with PND are also considered high-risk precancerous lesions [20].

PND presents a significant cancer risk, making the diagnosis and treatment a persistent challenge for breast surgeons. While the characteristics of PND are well documented in the literature, comparative clinical and risk factor analyses across different pathologic subtypes are lacking. In this study, we aim to compare and summarize the clinical characteristics of 375 PND patients across four pathological types: non-neoplastic lesions, benign neoplastic lesions, precancerous lesions, and breast cancer, and analyze independent risk factors for breast cancer and precancerous lesions.

## 2. Methods

### 2.1. Study Design

The inclusion criteria for this study encompassed patients with nipple discharge who had undergone postoperative pathology. Patients presenting only imaging results or a clinical diagnosis were excluded. Biopsy and postoperative pathological examination results were considered the definitive diagnostic standards. A research assistant, unaware of the study hypothesis, extracted basic information, medical findings, and pathology results from the electronic medical record database at the Beijing Hospital of Traditional Chinese Medicine, using “nipple discharge” as the index term. Medical records and pathology were reviewed by two experienced doctors from the Galactophore department and one experienced pathologist. Additionally, a fourth doctor collected data for analysis. From July 2014 to July 2022, 375 patients whose records met the inclusion and exclusion criteria were included in the study.

### 2.2. Statistical Analysis

Data analysis was conducted by SPSS 26.0 software. The significance level was defined as *p* < 0.05. For univariate analysis, the *χ*^2^ test was employed. Clinically significant factors were identified and used as independent variables. Statistically significant variables were further evaluated using multivariate logistic regression analysis, which included the calculation of odds ratios (ORs) and 95% confidence intervals (CIs) [21].

## 3. Results

### 3.1. Characteristics of Study Population

All 375 patients with PND were females from China.  Table 1 summarizes the demographic characteristics of all patients. Of these, 335 (89.33%) were married and 40 (10.67%) were unmarried. The age distribution was as follows: under 45 years, 229 (61.07%); 45 years and older, 146 (38.93%). The duration of onset for 114 patients was as follows: ≤ 6 months in 70 (61.40%), 6–12 months in 19 (16.67%), and > 12 months in 25 (21.93%). The shortest duration was 1 day (4 cases), the longest 17 years (1 case), with an average history of approximately 16 months.

**Figure 1:**
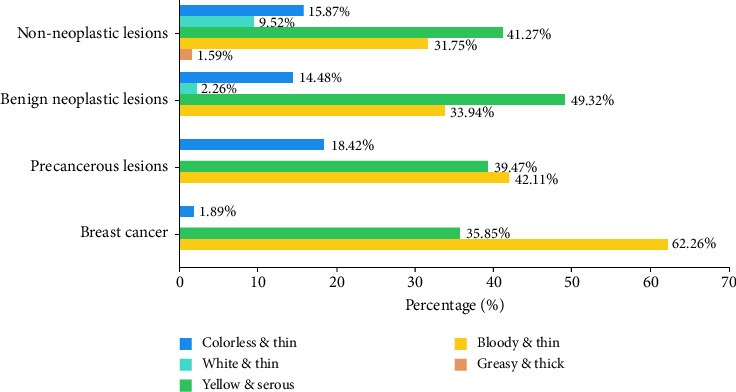
Discharge color in each pathological classification.

Table 2 summarizes the clinical characteristics and pathology of 375 patients with PND. Among these, 276 (73.60%) presented with unilateral discharge, while 99 (26.40%) exhibited bilateral discharge. Of the patients, 317 (84.53%) had discharge from a single orifice, and 58 (15.47%) from multiple orifices. The discharge color varied: 50 patients (13.33%) had colorless, 11 (2.93%) white, 169 (45.07%) yellow, 144 (38.40%) bloody, and 1 (0.27%) greasy. Discharge color in each pathological classification refers to Figure 1. Discharge volume was as follows: 172 patients (45.87%) had a large amount, 121 (32.27%) medium, and 82 (21.87%) small. Of the 327 patients who underwent breast ductoscopy, 305 (93.27%) presented with a visible mass, and 22 (6.73%) showed no mass, suggesting intraductal inflammation. The 375 PND patients were categorized into four groups based on postoperative pathology: non-neoplastic lesions 63 (16.80%), benign neoplastic lesions 221 (58.93%), precancerous lesions 38 (10.13%), and breast cancer 53 (14.13%).

**Figure 2:**
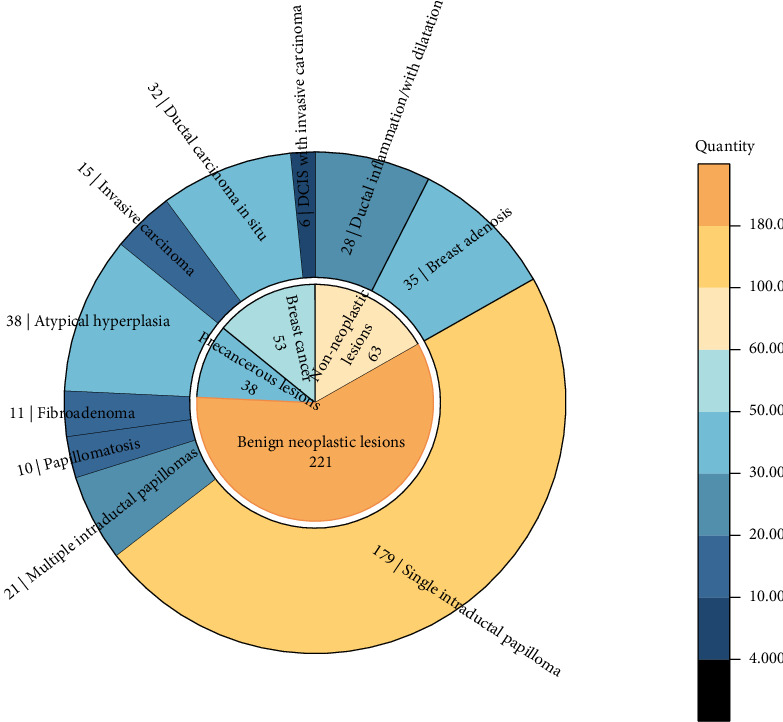
Pathological classification of PND.

Upon our investigation, we found that there are several main categories (Figure 2): ductal inflammation/with dilatation (7.47%), breast adenosis (9.33%), single intraductal papilloma (47.73%), multiple intraductal papillomas (5.60%), papillomatosis (2.67%), fibroadenoma (2.93%), atypical hyperplasia (10.13%), invasive carcinoma (4%), DCIS (8.53%), and DCIS with invasive carcinoma (1.60%).

**Figure 3:**
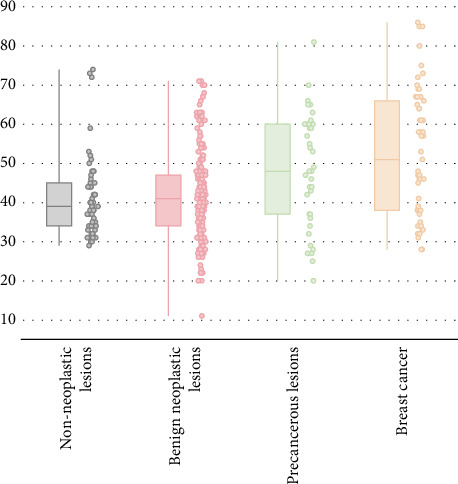
Age distribution of patients with PND by pathologic type (years).

Table 3 and Figure 3 present the ages of patients across the four pathology groups. The median age of the 375 patients was 42.00 years (mean age 43.94 years, range 11–86 years), and age was not normally distributed. The median ages of the groups were as follows: non-neoplastic lesions, 39 years (mean 40.92, range 29–74); benign neoplastic lesions, 41 years (mean 41.97, range 11–71); precancerous lesions, 48 years (mean 48.29, range 20–81); and breast cancer, 51 years (mean 52.60, range 28–86). Correspondingly, as the severity of the pathological diagnosis increases, so does the median age.

### 3.2. Relationship Between Pathologic and Clinical Factors

Table 4 details the correlation between pathological diagnoses and various clinical factors. Non-neoplastic and benign neoplastic lesions showed a high incidence rate in individuals under 45 years of age, while precancerous lesions and breast cancer were more common in those over 45 years of age (*p* < 0.01). Besides advanced age, discharge color was a significant factor in distinguishing between different pathological findings (*p* < 0.01). Bloody discharge was associated with a significantly higher incidence of breast cancer and precancerous lesions compared to non-bloody discharges, including yellow serous discharge and colorless types. Yellow serous discharge showed a significantly higher incidence of benign neoplastic lesions and non-neoplastic lesions compared to other discharge types. White discharge was exclusive to non-neoplastic lesions and benign neoplastic diseases, not observed in precancerous lesions and breast cancer. Greasy discharge was unique to intraductal inflammation. The incidence of benign neoplastic lesions in the group with colorless discharge was significantly higher than that of breast cancer. When categorizing bloody discharge into brown and bright-red subtypes for detailed analysis, no significant differences were noted across various pathological types (*p* > 0.05). A total of 327 patients underwent ductoscopy, resulting in significant findings in all breast cancer cases, 1 false negative in precancerous lesions, and 10 false negatives in benign neoplastic lesions. These results indicate a higher diagnostic rate of ductoscopy for breast cancer and precancerous lesions (*p* < 0.01). In contrast, factors such as marital status, medical history, unilateral or bilateral discharge, number of orifices, and discharge volume did not significantly correlate with the pathologic types (*p* > 0.05).

### 3.3. Multivariate Logistic Regression Analysis

**Figure 4:**
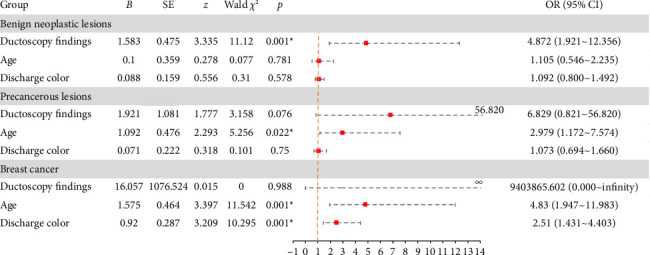
Multivariate logistic regression analysis of PND.

A multivariate logistic regression model was constructed, incorporating factors such as “Age,” “Discharge Color,” and “Ductoscopy Findings” (Figure 4).

a. Abnormal “Ductoscopy Findings” significantly impacted benign neoplastic lesions (OR = 4.872, 95% CI 1.921–12.356, *p*=0.001);
b. “Age” had a significant statistical effect on precancerous lesions (OR = 2.979, 95% CI 1.172–7.574, *p*=0.022);
c. “Age” significantly affected breast cancer (OR = 4.830, 95% CI 1.947–11.983, *p*=0.001), as did “Discharge Color” (OR = 2.510, 95% CI 1.431–4.403, *p*=0.001).

## 4. Discussion

### 4.1. Age

Age is acknowledged as a crucial factor in the consideration of PND. Multivariate regression analysis identified “Age” as an important independent risk factor for breast cancer and precancerous lesions with nipple discharge, in contrast to pathologic discharge from non-neoplastic lesions. When age increases from “< 45” to “≥ 45,” the risk of progression from “non-neoplastic” to “precancerous” lesions multiplies by 2.979. Additionally, the risk of transitioning from “non-neoplastic lesions” to “breast cancer” escalates by 4.830 times.

Women diagnosed with malignancies were significantly older, often postmenopausal, compared to those with benign histology [22]. Seltzer's review of 10,000 breast disease cases revealed that the majority (68%) of patients were under 50 years old [23]. Among these patients, only 4% under 50 years old were diagnosed with breast cancer, while 17% aged 50 or older had the disease. It is now acknowledged that PND patients over 50 years of age have an elevated risk of breast cancer [24, 25]. Nevertheless, our study highlights the age of 45 as a pivotal milestone in the development of precancerous lesions and breast cancer in women. The study found that non-neoplastic and benign neoplastic lesions were more prevalent in women under 45, whereas precancerous and breast cancer lesions were more common in those aged 45 and above. Breast cancer incidence stood at 33.96% in patients < 45 years and 66.04% in those ≥ 45 years. With malignancy progression in pathology, there is an increase in median age, underscoring the need for increased vigilance in those over 45, where risks for breast cancer and precancerous lesions escalate.

### 4.2. Discharge

#### 4.2.1. Discharge Color

Discharge color is also deemed a critical factor in PND assessment. Multivariate regression analysis revealed that discharge color is a significant independent risk factor for breast cancer in PND, contrasting with PND arising from non-neoplastic lesions.

Bloody discharge was associated with a significantly higher incidence of breast cancer and precancerous lesions compared to non-bloody discharges, including yellow and colorless types. Yellow serous discharge showed a significantly higher incidence of benign neoplastic lesions and non-neoplastic lesions compared to other discharge types. White discharge was exclusive to non-neoplastic lesions and benign neoplastic diseases, not observed in precancerous lesions and breast cancer. Greasy discharge was unique to intraductal inflammation.

In this study, the most prevalent PND colors were yellow serous (45.07%) and bloody (38.40%). Among these, non-neoplastic and benign neoplastic lesions most commonly presented with yellow serous discharge, followed by bloody discharge. In this study, the most prevalent PND colors were yellow serous (45.07%) and bloody (38.40%). Among these, non-neoplastic and benign neoplastic lesions most commonly presented with yellow serous discharge, followed by bloody discharge. Of the precancerous lesions, 7 cases (7/38) presented with colorless discharge, and 1 case (1/53) of breast cancer also had colorless discharge. White serous discharge was absent in both precancerous lesions and breast cancer cases. This suggests that the risk of malignancy is highest with bloody discharge, followed by yellow serous, and then colorless discharge. Given the absence of breast cancer or precancerous lesions with milky serous discharge in this study, the malignancy risk associated with this discharge type remains uncertain.

Chen et al. discovered that patients with hemorrhagic discharge had an increased risk of developing breast cancer compared to those with other discharge types [26]. Wang Fuwen's study revealed a significantly higher rate of precancerous lesions/malignancy in the bloody discharge group compared to the non-bloody discharge group, with all breast cancer patients exhibiting bloody or brown discharge, except for one patient with atypical hyperplasia who had non-bloody discharge [27]. This similarly indicates a heightened risk of malignancy associated with bloody discharge. In our study, the incidence of benign neoplasms in cases of bloody discharge was significantly higher than that of breast cancer, reflecting the inherently high incidence of benign neoplasms. Additionally, the incidence of breast cancer was notably higher in the bloody discharge group compared to non-bloody discharge groups, including those with yellow serous and colorless discharge. Furthermore, in Table 4, bloody discharge was categorized into brown (79.17%) and bright red (20.83%) for detailed analysis, revealing no significant difference among various pathological types. This suggests that the risk associated with bloody discharge is independent of its specific color, with the risk of brown comparable to bright red.

Additionally, this study included a case of unilateral uniportal yellowish-green greasy discharge in a 34-year-old patient, with a medium volume of discharge. Ductoscopy suggested “mastitis,” while ultrasound revealed a “cystic solid nodule, 1.6 × 0.8 cm, with clear borders along the ductal route.” Postoperative pathology indicated “breast cyst formation with surrounding inflammatory cell infiltration and ductal dilatation,” leading to the conclusion that the yellow-green greasy discharge was likely due to ductal inflammation.

#### 4.2.2. Discharge Duct

Prior studies have shown that PND usually presents as uniportal and unilateral. However, the risk of malignancy also exists with multiple discharges. Peripheral papillomas often manifest as multiple and bilateral and are linked to an increased risk of malignancy [28]. In our study, 16.98% of breast cancer cases (9/53) involved multiple ducts: 6 with yellow serous discharge and 3 with bloody discharge. Patient 1: A 28-year-old unmarried woman with a family history of breast cancer, presenting bloody discharge from both breasts, multiple orifices on the right, and a single orifice on the left. Postoperative pathology revealed invasive breast cancer in the left breast (tumor size 0.6 × 0.4 cm) and intraductal papilloma with DCIS; in the right breast, intraductal papilloma with DCIS. Patient 2: A 69-year-old married woman, with multiple orifices in both breasts exuding yellowish plasma discharge, one orifice in the left breast had bloody discharge. Postoperative pathology indicated left breast intraductal papillary carcinoma (G1), multifocal, maximum size 0.7 cm; right breast with intraductal papillary carcinoma (G1) and DCIS (G1), multifocal, maximum size 0.3 cm.

#### 4.2.3. Discharge Volume

Thirty-three percent of breast cancer patients and 25% with highly atypical hyperplasia exhibited occult ductal bleeding, leading to the conclusion that the absence of nipple bleeding does not rule out breast cancer [29]. This study also indicated a correlation between the volume of ductal bleeding and an increased risk of breast cancer or highly atypical hyperplasia. In our study, discharge volume was categorized as large, medium, or small. “Small” was defined as no spontaneous discharge or ≤ 1 mm per nipple squeeze, occasionally no discharge; “medium” as 1∼3 mm per squeeze; and “large” as > 3 mm or jet-like discharge. Our study revealed that breast cancer patients predominantly had “medium” or “large” fluid discharges, yet no statistically significant difference was observed in discharge volumes among different pathological types.

### 4.3. Ductoscopy

Interventional ductoscopy can detect (pre)malignant lesions and can prevent unnecessary diagnostic surgical procedures in 2 of 3 patients suffering from PND [30]. There was a statistically significant effect of abnormal ductoscopy findings on benign neoplastic lesions (OR = 4.872, 95% CI 1.921∼12.356, *p*=0.001). This suggests that when the change in ductoscopy findings was from “inflammation” to “mass,” there was a 4.872-fold increase in the risk of changing from a “non-neoplastic lesion” to a “benign neoplastic lesion.” When ductoscopy was successful, the odds of finding a significant lesion on ductoscopy were increased 7.5-fold if classical PND was present (95% CI 1.5–38.5) [31]. The use of ductoscopy has shown improvement in selecting patients for duct excision when compared to conventional clinical and imaging criteria.

Ductoscopy, especially effective for central intraductal lesions, is a highly sensitive diagnostic method for detecting lesions in nipple discharge cases not identifiable by ultrasound or mammography. In mammography, cancers are frequently obscured by overlapping dense breast tissue, leading to the frequent omission of small or noncalcified lesions. As a result, the false-negative rate remains significant [32]. In contrast, ductoscopy is a minimally invasive technique that allows direct visualization of the intraductal lumen, offering a more intuitive and accurate method for early detection and localization of ductal lesions.

However, ductoscopy and other imaging modalities have inherent limitations, as they predominantly assess morphological features and do not adequately reflect the biological behavior of lesions. Consequently, the safety of ductoscopy in women with normal findings remains uncertain and requires further validation through larger cohort studies and extended follow-up.

Studies have shown that integrating artificial intelligence (AI) into mammography significantly reduces both false-positive and false-negative rates, and decreases the second reader's workload by up to 88% [33]. Thus, robust evaluation of AI systems has enhanced both the accuracy and efficiency of breast cancer screening in clinical settings. Notably, the future integration of ductoscopic images into AI-assisted diagnostic systems—leveraging ductoscopy's high-resolution, direct visualization with AI's capabilities in image recognition and lesion classification—could significantly enhance the sensitivity and consistency of intraductal microlesion detection. This multimodal, AI-driven diagnostic strategy holds significant promise for advancing precision screening and early intervention in breast diseases.

In recent years, the emergence of radiomics has opened new avenues for the accurate detection of breast cancer [34]. This technique involves extracting high-throughput quantitative features—such as texture, shape, and intensity—from conventional imaging modalities (e.g., MRI, ultrasound, or mammography), followed by data modeling using machine learning or deep learning algorithms. This approach is expected to enhance the detection of subtle lesions and improve malignancy risk prediction, thereby significantly boosting the diagnostic performance of imaging for intraductal breast lesions.

### 4.4. Limitations

Firstly, imaging studies were excluded from this study due to their high false-negative rate. Imaging techniques like mammograms, tomosynthesis, and high-resolution ultrasound are used to investigate potential intraductal lesions [11, 35]. However, these methods have limited value, and magnetic resonance imaging also fails to identify all cases of intraductal neoplasia [31]. Even when ultrasound and mammography results are negative, the risk of malignancy remains at approximately 5%–8% [36, 37]. Secondly, future studies could benefit from incorporating tumor marker testing to enhance the accuracy of breast cancer diagnosis.

## 5. Conclusions

All cases of pathologic discharge exhibited a consistent pattern: higher lesion rates in married, unilateral, and uniportal cases, larger discharge volumes, and a disease duration typically ≤ 6 months. However, significant differences were noted in terms of age and the color of the discharge. Age, an important independent risk factor, becomes particularly significant when exceeding 45 years, correlating with a notably higher risk of breast cancer and precancerous lesions. Concerning discharge color, bloody discharge poses the highest malignancy risk, with the clinical risk of brown discharge comparable to bright red. Yellow serous discharge follows, then colorless discharge in terms of risk. Ductoscopy boasts a higher detection rate in identifying breast cancer and precancerous lesions.

## Figures and Tables

**Table 1:** Demographic characteristics of the study population, *n* (%).

Characteristics	Cases (*n* = 375)
Sex	
Female	375 (100.00)
Duration of onset (*n* = 114)	
≤ 6 months	70 (61.40)
≥ 6 and ≤ 12 months	19 (16.67)
> 12 months	25 (21.93)
Age, mean ± SD	43.94 ± 12.72
< 45 years	229 (61.07)
≥ 45 years	146 (38.93)
Marital status	
Married	335 (89.33)
Single	40 (10.67)

**Table 2:** Clinical characteristics and pathology of nipple discharge, *n* (%).

Characteristics	Cases (*n* = 375)
Affected breast	
Unilateral	276 (73.60)
Bilateral	99 (26.40)
Discharge duct	
Single	317 (84.53)
Multiple	58 (15.47)
Discharge color	
Colorless and thin	50 (13.33)
White and thin	11 (2.93)
Yellow and serous	169 (45.07)
Bloody and thin	144 (38.40)
Greasy and thick	1 (0.27)
Discharge amount	
Large	172 (45.87)
Medium	121 (32.27)
Small	82 (21.87)
Ductoscopy findings (*n* = 327)	
Inflammation	22 (6.73)
Mass	305 (93.27)
Pathological type	
Non-neoplastic lesions	63 (16.80)
Benign neoplastic lesions	221 (58.93)
Precancerous lesions	38 (10.13)
Breast cancer	53 (14.13)

**Table 3:** Age distribution of patients with PND by pathologic type (years).

Groups	*n* (%)	Min	Max	Mean ± SD	Median
Total	375 (100.00)	11.00	86.00	43.94 ± 12.72	42.00
Non-neoplastic lesions	63 (16.80)	29.00	74.00	40.92 ± 9.70	39.00
Benign neoplastic lesions	221 (58.93)	11.00	71.00	41.97 ± 10.98	41.00
Precancerous lesions	38 (10.13)	20.00	81.00	48.29 ± 14.50	48.00
Breast cancer	53 (14.13)	28.00	86.00	52.60 ± 16.54	51.00

**Table 4:** Comparisons of clinical characteristics among different pathological types.

Variables	Pathological type, *n* (%)				Total	*p*-value
	Non-neoplastic lesions	Benign neoplastic lesions	Precancerous lesions	Breast cancer		
Marital status						0.48
Married	59 (93.65)	196 (88.69)	32 (84.21)	48 (90.57)	335 (89.33)	
Single	4 (6.35)	25 (11.31)	6 (15.79)	5 (9.43)	40 (10.67)	
Age (years)						0.001^∗^
< 45	47 (74.60)	148 (66.97)	16 (42.11)	18 (33.96)	229 (61.07)	
≥ 45	16 (25.40)	73 (33.03)	22 (57.89)	35 (66.04)	146 (38.93)	
Time of onset (*n* = 114)						0.93
≤ 6 months	14 (58.33)	43 (64.18)	3 (42.86)	10 (62.50)	70 (61.40)	
≥ 6 and ≤ 12 months	5 (20.83)	10 (14.93)	2 (28.57)	2 (12.50)	19 (16.67)	
> 12 months	5 (20.83)	14 (20.90)	2 (28.57)	4 (25.00)	25 (21.93)	
Affected breast						0.34
Unilateral	42 (66.67)	170 (76.92)	27 (71.05)	37 (69.81)	276 (73.60)	
Bilateral	21 (33.33)	51 (23.08)	11 (28.95)	16 (30.19)	99 (26.40)	
Discharge duct						0.38
Single	49 (77.78)	191 (86.43)	33 (86.84)	44 (83.02)	317 (84.53)	
Multiple	14 (22.22)	30 (13.57)	5 (13.16)	9 (16.98)	58 (15.47)	
Discharge color						0.001^∗^
Colorless and thin	10 (15.87)	32 (14.48)	7 (18.42)	1 (1.89)	50 (13.33)	
White and thin	6 (9.52)	5 (2.26)	0 (0.00)	0 (0.00)	11 (2.93)	
Yellow and serous	26 (41.27)	109 (49.32)	15 (39.47)	19 (35.85)	169 (45.07)	
Bloody and thin	20 (31.75)	75 (33.94)	16 (42.11)	33 (62.26)	144 (38.40)	
Greasy and thick	1 (1.59)	0 (0.00)	0 (0.00)	0 (0.00)	1 (0.27)	
Bloody discharge						0.60
Brown	18 (90.00)	58 (77.33)	13 (81.25)	25 (75.76)	114 (79.17)	
Bright red	2 (10.00)	17 (22.67)	3 (18.75)	8 (24.24)	30 (20.83)	
Discharge amount						0.75
Large	27 (42.86)	106 (47.96)	18 (47.37)	21 (39.62)	172 (45.87)	
Medium	19 (30.16)	68 (30.77)	12 (31.58)	22 (41.51)	121 (32.27)	
Small	17 (26.98)	47 (21.27)	8 (21.05)	10 (18.87)	82 (21.87)	
Ductoscopy findings (*n* = 327)						0.001^∗^
Inflammation	11 (21.15)	10 (5.10)	1 (2.94)	0 (0.00)	22 (6.73)	
Mass	41 (78.85)	186 (94.90)	33 (97.06)	45 (100.00)	305 (93.27)	

^∗^ *p* < 0.01.

## Data Availability

The data used to support the findings of this study are available from the corresponding author upon reasonable request.
